# Mask dependency of the lacrimal gland dose under whole brain radiotherapy when the six‐degrees of freedom couch is not available

**DOI:** 10.1002/acm2.14052

**Published:** 2023-05-31

**Authors:** Jai‐Woong Yoon, Me Young Kim, Soah Park, Kwang‐Ho Cheong, Sei‐Kwon Kang, Taeryool Koo, Tae Jin Han

**Affiliations:** ^1^ Department of Radiation Oncology Dongtan Sacred Heart Hospital Hwaseong Korea; ^2^ Department of Radiation Oncology Chuncheon Sacred Heart Hospital Hallym University College of Medicine Gangwon‐do Korea; ^3^ Department of Radiation Oncology Kangnam Sacred Heart Hospital Hallym University College of Medicine Seoul Korea; ^4^ Department of Radiation Oncology Hallym University Sacred Heart Hospital Hallym University College of Medicine Anyang Korea; ^5^ Department of Radiation Oncology Kangdong Sacred Heart Hospital Hallym University College of Medicine Seoul Korea

**Keywords:** 6D couch, dry eye syndrome, frameless stereotactic mask, lacrimal gland, rotational error, whole brain treatment

## Abstract

**Background:**

Dry eye syndrome has been recently reported in patients who underwent whole brain radiotherapy (WBRT). WBRT based on a couch with three‐degrees of freedom (3D) can occasionally be performed in which the rotational head motion is not corrected. This study assessed the dependency of the rotational errors on the mask and the dose variation of the lens and lacrimal gland in WBRT patients.

**Methods:**

Translational and rotational setup errors were obtained at the first treatment with cone‐beam CT (CBCT) for patients under WBRT and frameless stereotactic radiosurgery (SRS) (*n* = 20 each) immobilized using a conventional WB mask and an SRS mask with a bite block, respectively. For the CT sets of SRS cases, WBRT plans were generated for the study. To simulate the rotational error, rotated CT images were created with each rotational error, on which initial WBRT plans were copied and doses were recalculated. The lens and lacrimal gland doses with and without rotation errors were compared.

**Results:**

Despite similar translational setup errors for the two masks, the SRS mask showed a dramatic reduction in rotational errors compared to those of the WB mask. The errors varied within −2.9° to 2.9° and −1.2° to 0.7° for the WB and SRS masks, respectively. Accordingly, the SRS mask confined the change in the maximum lens dose, mean dose of the lacrimal gland, and lacrimal volume receiving 15 Gy to one‐third of those using the WB mask.

**Conclusion:**

When the six‐degrees of freedom (6D) couch is not available, the frameless SRS mask is beneficial to WBRT for the faithful treatment as it was planned.

## 1 INTRODUCTION

Whole brain radiotherapy (WBRT) has been one of the standard treatment options for multiple brain metastases, and is known to cause some side effects including fatigue, drowsiness, motor dysfunction, and communication deficits.^1^, ^2^ However, owing to the simplicity of the technique with a relatively low prescribed dose and the predominantly poor prognosis of patients, probable toxicity to normal organs has been noted with less caution. However, the advancement of treatments has significantly improved the prognosis of patients with WBRT, and the concern about radiation toxicity with the quality of life (QoL) has been increased naturally.^3^ Patients undergoing prophylactic cranial irradiation (PCI) warrant greater concern since it has been considered as the standard care to reduce the risk of brain metastases in patients with the small cell lung cancer.^4^

Recently, dry eye syndrome was reportedly associated with lacrimal gland dose.^5^, ^6^ Image guided radiotherapy (IGRT), particularly, a six‐degrees of freedom (6D) couch top plays a vital role in accurate treatment delivery by introducing a rotational correction in addition to the translational correction by the three‐degrees of freedom (3D) couch.^7^, ^8^ However, depending on the clinic, the 6D couch may not be always available and WBRT patients would be prepared for treatment using a 3D couch, which means that the probable rotational error is not corrected. This necessitates an assessment of the immobilization capabilities of whole brain masks.

In this study, for two groups of patients who underwent WBRT and frameless stereotactic radiosurgery (SRS) who were immobilized using a conventional WB mask and a frameless SRS mask, respectively, we retrospectively created WBRT plans with and without the rotational errors obtained at the first treatment of each patient and compared the dose variation for the lens and lacrimal gland.

## 2 METHODS

Translational and rotational setup errors were obtained for patients consecutively selected for WBRT and SRS cases (*n* = 20 each). For head immobilization, a conventional head only thermoplastic mask (Uniframe, CIVCO) was used for the WBRT. For SRS treatment, a frameless head mask (Fraxion; Elekta AB, Stockholm, Sweden) was applied, which provides a bite block for teeth fixation and a cushion between the patient's head and the head rest. The bite block was molded using a dental impression material, while the vacuum suction accompanied to prevent saliva secretion was not adopted. For the treatment planning, the beam isocenter was located near the center of the brain and at each center of the target in WBRT and SRS cases, respectively. For each treatment setup, cross‐hairs on the mask system were aligned to the room lasers and kV cone‐beam CT (CBCT) was obtained using the X‐ray volume imaging system XVI of Elekta. Following the rigid registration of the CBCT with the plan CT, the translational, and rotational setup errors were corrected by using a 6D couch, HexaPod of Elekta, in which the center of the registration was set at the beam isocenter. The translational and rotational setup errors at the first treatment of each patient were acquired and used for the subsequent creation of CT images with rotational errors.

Patient CT images were retrospectively reconstructed with 1‐mm slice thickness. For the WBRT plan for both patient groups, the delineated brain was expanded by 5 mm for the planning target volume, and the lens and lacrimal gland were contoured. The field‐in‐field technique with two lateral beams was used with a prescription of 30 Gy in 10 fractions. The dose to the lens was constrained to <5 Gy using multi‐leaf collimator blocking, and the intentional dose saving for the lacrimal glands was not considered. For the WBRT group, the isocenter was the same as the original treatment plan. For the SRS group, the beam isocenter was newly selected around the center of the brain. The dose grid for calculation was set to 1 mm. To simulate the dose variation of WBRT under the corrected translational error and uncorrected rotational error, CT images along with the delineated organs were rotated three‐dimensionally around the beam isocenter for each patient by the individual rotational errors acquired during the setup for the first treatment. Then, the parameters of the WBRT plan were copied to the rotated CT set, followed by dose recalculation. The Mahn–Whittney test was used for the statistical evaluation of the parameters.

## 3 RESULTS

**FIGURE 1:**
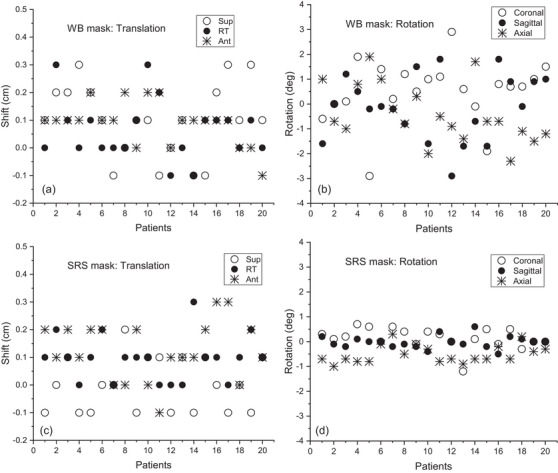
Patient setup errors obtained at the first treatment. (a) Translational and (b) rotational errors for the WB mask; (c) translational and (d) rotational errors for the SRS mask. The rotational errors for the SRS mask were greatly controlled than those for the WB mask. (Sup, Superior; RT, Right; Ant, Anterior).

For the lens volume, the mean with the standard deviations was 0.14 ± 0.04 and 0.13 ± 0.04 cm^3^ for the WB and SRS masks, respectively (*P* = 0.226). In the case of the lacrimal gland, it was 0.45 ± 0.25 and 0.51 ± 0.18 cm^3^ for the WB and SRS masks, respectively (*P* = 0.097). The volumes of lens and lacrimal gland were similar for both masks. The translational and rotational setup errors at the first treatment for each patient are shown in Figure 1. The superior‐inferior (SI), left‐right (LR), and anterior‐posterior (AP) translational errors depicted a similar distribution for both masks, ranged from −0.1 to 0.3 cm. Meanwhile, the SRS masks displayed a dramatic reduction in the rotational errors compared to those of WB masks; the errors varied within −2.9° to 2.9° and −1.2° to 0.7° for the WB and SRS masks, respectively. For the related mean and standard deviations (Table 1), the magnitudes of errors were considered since we focused on the deviation from the origin. No difference was observed in the translational errors of LR and AP for both masks, that is, 0.085 ± 0.09 (WB) and 0.10 ± 0.08 cm (SRS) (*P* = 0.59) for LR direction and 0.10 ± 0.06 (WB) and 0.13 ± 0.10 cm (SRS) (*P* = 0.20) for AP direction. For SI, the mean deviations were smaller for SRS mask, that is, 0.14 ± 0.09 and 0.075 ± 0.06 cm for the WB and SRS masks, respectively (*P* = 0.02). In the rotational errors, SRS mask produced significantly smaller values (*P* < 0.001), that is, 1.06 ± 0.84° (WB) and 0.34 ± 0.30° (SRS) for roll direction, 1.06 ± 0.76° (WB) and 0.21 ± 0.18° (SRS) for pitch, and 1.09 ± 0.56° (WB) and 0.54 ± 0.28° (SRS) for yaw, respectively.

**TABLE 1:** A comparison of the translational and rotational setup errors for the WB and SRS masks.

		WB mask	SRS mask	*P*
Translation (cm)	SI	0.14 ± 0.09	0.075 ± 0.06	0.02
	LR	0.085 ± 0.09	0.10 ± 0.08	0.59
	AP	0.10 ± 0.06	0.13 ± 0.10	0.20
Rotation (°)	Roll	1.06 ± 0.84	0.34 ± 0.30	<0.001
	Pitch	1.06 ± 0.76	0.21 ± 0.18	<0.001
	Yaw	1.09 ± 0.56	0.54 ± 0.28	<0.001

*Note*: The errors were calculated in absolute values.

**Abbreviations:** AP, Anterior‐Posterior; LR, Left‐Right; SI, Superior‐Inferior.

For the simulation accuracy of the rotation of the delineated organs, we determined the volume variation of the lacrimal gland as an example before and after rotation. The difference was 0.7% with a standard deviation of 1.0%, and the two distributions were not significantly different (*P* = 0.98). Table 2 summarizes the mean and standard deviations of the dose and volume differences in the magnitude caused by rotational error for the WB and SRS masks. For the lens and lacrimal glands, the volumes of the left and right were combined for the analysis. Regarding the rotational error, the maximum lens dose varied by 19.5 ± 18.2 cGy (WB) and 7.0 ± 8.4 cGy (SRS) (*P* < 0.001). For the mean dose of the lacrimal gland, the change was 206.3 ± 331.2 cGy (WB) and 54.9 ± 52.0 cGy (SRS) (*P* < 0.001). In the case of V15 of the lacrimal glands, the change was 7.3 ± 9.4% volume (WB) and 2.5 ± 2.3% volume (SRS) (*P* = 0.001).

**TABLE 2:** Dose differences of the lens and lacrimal glands before and after rotation.

		WB mask	SRS mask	*P*
Lens	D_max_ (cGy)	19.5 ± 18.2	7.0 ± 8.4	<0.001
Lacrimal	D_mean_ (cGy)	206.3 ± 331.2	54.9 ± 52.0	<0.001
	V15 (%volume)	7.3 ± 9.4	2.5 ± 2.3	0.001

*Note*: Lens maximum dose (D_max_) and lacrimal mean dose (D_mean_) and lacrimal volume receiving 15 Gy (V15 %volume).

## 4 DISCUSSION

The similar translational errors for the WB and SRS masks could be expected since the translational setup environment was identical for both masks, in which cross‐hairs on the mask surface were aligned to the room lasers. The rotational errors were different. Unlike the WB mask, the SRS mask included a bite block grasping the patient's teeth, thereby reducing the probability of head rotation. Remarkably, the rotational errors using the SRS mask were less than half of those obtained using the WB mask in all directions (Table 1). The plan comparison before and after CT rotation demonstrated that the SRS mask ensured a significantly reduced deviation from those of the initial plan. The SRS mask warranted the variation of the lens and lacrimal glands doses less than a third of those of the WB mask (Table 2).

PinPoint (Aktina Medical, NY), an alternative bite‐block SRS mask, designed practically similar to our SRS mask, was assessed for the setup accuracy, and generated results similar to that of ours.^9^ In a study for patients undergoing intracranical fractionated stereotactic radiotherapy, Uniframe, the conventional WB mask, displayed more than three times of rotational errors than those of PinPoint in all directions. The better positional stability by PinPoint was maintained in intrafractional motion, such that the motion extent >1.5 mm was not observed for PinPoint; however, it amounted to 8% of all fractions for the Uniframe application. For the rotational error, PinPoint demonstrated significantly lower mean and standard deviation values in all directions.^9^, ^10^ The head fixation ability of the SRS mask during setup was also reported for the BrainLAB (BrainLAB, Germany) frameless mask, in which the mean rotational errors of −0.10 ± 1.03°, 0.23 ± 0.82°, and −0.09 ± 0.72° for the vertical, longitudinal, and lateral directions, respectively. Therefore, the SRS mask was useful for head immobilization, both in setup and during treatment.

Recently, the self‐evaluated dry eye syndrome was reported as an acute toxicity of WBRT.^6^ Approximately one‐third of the patients with WBRT developed dry eye syndrome, and following 1 month of treatment completion, lacrimal volumes of higher doses receiving 10 Gy (V10Gy) and 20 Gy (V20Gy) were associated with more severe symptoms. Therefore, to improve the QoL of WBRT patients, more attention was demanded to reduce the lacrimal dose that caused this acute side effect. Both lenses and lacrimal glands are susceptible to large variation in beam exposure under a small change in the head position during WBRT. The robotic 6D couch is essential for a minimal deviation from the optimized.^7^, ^11^, ^12^ When the 3D couch with a CBCT is an only option in the clinic without a 6D couch, the SRS mask rather than the conventional WB mask is recommended for reducing the rotational head motion of patients with WBRT for the reliable sparing of normal organs.

This study had few limitations. First, in contrast to the WBRT cases, the rotation center for creating the rotated CT set for the SRS case was different from the registration center of the planned CT to the CBCT; that is, the parameters for the rotational errors were not acquired at the new center for the images. However, we assumed that the change in the registration center would be reflected in the translational registration and the rotational errors would be maintained. Second, the delineation of the lacrimal glands is personnel dependent and vulnerable to large variations owing to its small volume. However, exact definition of the lacrimal glands was not considered in this study because we aimed to identify the relative change in the dose parameters under positional variations during the setup.

In conclusion, when the 6D couch is not available, the frameless stereotactic head mask could reduce the rotational setup errors by more than half of those by the conventional WB mask with the translational error being corrected by the 3D couch. Therefore, based on the treatment plan, the SRS mask could confine the dose deviations of the lens and lacrimal glands to one‐third of those by the WB mask.

## AUTHOR CONTRIBUTIONS

Jai‐Woong Yoon and Sei‐Kwon Kang designed the study including literature research and wrote the manuscript. Soah Park, Kwang‐Ho Cheong, and Me Young Kim performed simulation and extracted data. Sei‐Kwon Kang, Taeryool Koo, and Tae Jin Han assessed the data. All authors read and approved the final manuscript.

## CONFLICT OF INTEREST STATEMENT

No conflict of interest.
